# Serologic features of cohorts with variable genetic risk for systemic lupus erythematosus

**DOI:** 10.1186/s10020-018-0019-4

**Published:** 2018-06-01

**Authors:** Jyotsna Bhattacharya, Karalyn Pappas, Bahtiyar Toz, Cynthia Aranow, Meggan Mackay, Peter K. Gregersen, Ogobara Doumbo, Abdel Kader Traore, Martin L. Lesser, Maureen McMahon, Tammy Utset, Earl Silverman, Deborah Levy, William J. McCune, Meenakshi Jolly, Daniel Wallace, Michael Weisman, Juanita Romero-Diaz, Betty Diamond

**Affiliations:** 1 The Feinstein Institute for Medical Research, Center for Autoimmune, Musculoskeletal and Hematopoietic Diseases, 350 Community Dr, Manhasset, NY 11030 USA; 2 Department of Statistical Science, Cornell University, Ithaca, NY USA; 3 Department of Internal Medicine, Istanbul University, Istanbul, Turkey; 4 The Feinstein Institute for Medical Research, Center for Genomics and Human Genetics, Manhasset, NY USA; 5 Malaria Research and Training Center, Bamako, Mali; 6 Deputy of the Department of Internal Medicine, University Hospital, Bamako, Mali; 7 The Feinstein Institute for Medical Research, Center of Biostatistics Unit Manhasset, Manhasset, NY USA; 8 UCLA David Geffen School of Medicine, Los Angeles, CA 90095 USA; 9 University of Chicago Medical Center, Chicago, IL USA; 10 Hospital for Sick Children, University of Toronto, Toronto, ON M5G 1X8 Canada; 11 University of Michigan, Ann Arbor, MI 48109 USA; 12 Rush University Medical Center, Chicago, IL 60612 USA; 13 Cedars Sinai Medical Center, Los Angeles, CA 90048 USA; 14 Instituto Nacional de Ciencias Medicas y Nutrician Salvador Zubiran, Mexico City, Mexico

## Abstract

**Background:**

Systemic lupus erythematosus (SLE) is an autoimmune disease with genetic, hormonal, and environmental influences. In Western Europe and North America, individuals of West African descent have a 3–4 fold greater incidence of SLE than Caucasians. Paradoxically, West Africans in sub-Saharan Africa appear to have a low incidence of SLE, and some studies suggest a milder disease with less nephritis. In this study, we analyzed sera from African American female SLE patients and four other cohorts, one with SLE and others with varying degrees of risk for SLE in order to identify serologic factors that might correlate with risk of or protection against SLE.

**Methods:**

Our cohorts included West African women with previous malaria infection assumed to be protected from development of SLE, clinically unaffected sisters of SLE patients with high risk of developing SLE, healthy African American women with intermediate risk, healthy Caucasian women with low risk of developing SLE, and women with a diagnosis of SLE. We developed a lupus risk index (LRI) based on titers of IgM and IgG anti-double stranded DNA antibodies and levels of C1q.

**Results:**

The risk index was highest in SLE patients; second highest in unaffected sisters of SLE patients; third highest in healthy African-American women and lowest in healthy Caucasian women and malaria-exposed West African women.

**Conclusion:**

This risk index may be useful in early interventions to prevent SLE. In addition, it suggests new therapeutic approaches for the treatment of SLE.

## Background

Systemic lupus erythematosus (SLE) is a chronic systemic autoimmune disease characterized by defects in B cell tolerance leading to the production of multiple autoantibodies. In particular, SLE is characterized by high affinity IgG anti-nuclear autoantibodies including anti-double stranded (ds) DNA antibodies.

Anti-dsDNA antibodies are found in 70% of patients, are pathogenic and are frequently used to monitor disease activity (Pavlovic et al. 2010; Linnik et al. 2005). Published data demonstrate a ‘preclinical’ period of disease characterized by the presence of IgG autoantibodies with increasing titers and number of auto- specificities heralding the onset of clinical SLE (Deane and El-Gabalawy 2014; Arbuckle et al. 2003). However, reports of elevated autoantibody titers in first degree relatives suggest that the presence of autoantibodies alone does not confer disease (Ramos et al. 2010).

While the etiology SLE is not known, data suggest that susceptibility requires both a genetic predisposition and environmental triggers. The genetic predisposition is highlighted by the observed familial clustering of SLE and a concordance rate of approximately 30% in identical twins. Over 50 risk alleles for SLE have been identified and disease severity and age of onset relates, in part, to the number of risk alleles present in an individual (Teruel and Alarcon-Riquelme 2016). Disease is 8–10 times more prevalent in women than men and 3–4 times more prevalent in women of African descent in Europe or North America than in Caucasian women (Gilkeson et al. 2011). In Caribbean populations, an increasing number of African genes rather than genetic admixture is a risk factor for disease (Molokhia et al. 2003; Molokhia and McKeigue 2000). The prevalence of SLE in West African women is not fully established, but several studies have suggested a lower prevalence in African countries (George and Ogunbiyi 2005; McGill and Oyoo 2002; Molokhia et al. 2001). Moreover, disease manifestations appear to be less severe in West African patients, with a lower incidence of renal disease (Zomalheto et al. 2014). It is reasonable to assume that the genetic predisposition to SLE is at least as high in West Africans as in African-Americans and Afro-Caribbeans and the discrepancy in disease prevalence reflects the impact of environmental factors (Molokhia et al. 2001).

Malaria, an endemic infection in sub-Saharan Africa, has long been suggested to mitigate the impact of SLE (Greenwood 1968). That malaria protects against development of SLE has been clearly demonstrated in spontaneously lupus-prone mice (Greenwood et al. 1970). Because it is frequently fatal, it likely has exerted significant pressure on the genome, resulting in the retention of alleles that diminish the severity of infection. Several risk alleles for SLE protect against severe malaria infection. The *FcRllb* risk allele for SLE (T232) leads to a non-functional molecule which cannot move through the plasma membrane to associate with the B cell receptor (Floto et al. 2005). Decreased inhibitory function associated with this risk allele results in increased B cell and myeloid cell activation. While this may increase risk for SLE, it can be beneficial for a response to infection. In humans, *FcRllb* T232 increases phagocytosis of *P. falciparum* by monocyte-derived macrophages in vitro (Clatworthy et al. 2007). Moreover, *FcRllb*-deficient mice are resistant to severe disease following infection with Plasmodium Chabaudi (Clatworthy et al. 2007). Notably, polymorphisms predisposing to low *TNF* levels protect against cerebral malaria. Several lupus-prone strains show reduced levels attributable to a promoter region polymorphism in the *NZB*, *BXSB* and *MRL* strains. (Jiang et al. 1999; Pritchard et al. 2000) and administering *TNF* to these mice can prevent the onset of SLE.

The repertoire of immunocompetent B cells develops as a consequence of tolerance mechanisms that censor a majority of autoreactive B cells during their maturation process. Approximately 75% of immature B cells have an autoreactive BCR compared to 20% of naïve immunocompetent B cells (Hoffman et al. 2016). These B cells are critical for immune homeostasis as they produce IgM antibodies capable of binding to and removing apoptotic debris in a non-immunogenic fashion (Gronwall et al. 2012). Lack of these autoreactive IgM antibodies results in uptake of apoptotic material in dendritic cells (DCs) and DC activation (Ehrenstein et al. 2000). In *NZB/W* lupus-prone mice, production of pathogenic IgG anti-dsDNA autoantibodies coincides with diminished production of IgM autoantibodies, and administration of IgM anti-dsDNA autoantibodies prevents development of renal disease in mice (Werwitzke et al. 2005).

Although malaria infection may protect against the development of SLE in spontaneous murine models of SLE, an association between malarial infection and autoantibodies is well recognized (Daniel-Ribeiro and Zanini 2000). Many of the autoantibodies present in malaria patients are IgM and are not known to be pathogenic (Wozencraft et al. 1990). The ability of IgM autoantibodies to maintain immune quiescence occurs through a C1q dependent mechanism (Gronwall and Silverman 2014).

C1q is a complement component that is important in clearance of apoptotic debris and promotes immune tolerance through regulation of immune cell differentiation and cytokine release (Son et al. 2015). Ninety percent of individuals with severe hereditary C1q deficiency have SLE (Manderson et al. 2004).

We hypothesized that an enhanced ratio of IgG:IgM anti-DNA antibodies and a diminished level of C1q would predispose to SLE. We further hypothesized that exposure to malaria results in increased titers of protective IgM autoantibodies and increases in C1q that retard or prevent onset of SLE in genetically predisposed individuals.

We, therefore, evaluated IgM and IgG anti-dsDNA antibody titers and assessed C1q levels in women with varying risk for SLE based on genetic risk and malaria exposure: African-American SLE patients (SLE); healthy Caucasian women (CHC); healthy African-American women (AAHC); unaffected sisters of SLE patients (SIS); and women from Mali with a history of malaria infection (MAL). We generated a lupus risk index (LRI) based on serum IgG:IgM anti-DNA antibody ratio and C1q level. The a priori hypothesis was that the LRI would be lowest in CHC, then increase through groups MAL, AAHC, SIS, and SLE, in that order. The development of an LRI may prove useful in following at risk individuals over time to identify those that may profit from early intervention and diagnosed SLE patients who might be at risk for an impending flare.

## Methods

### Samples

Serum samples were obtained from 40 Malian women, (MAL) aged 18 to 65. Inclusion criteria included a known history of malaria infection, no history of autoimmune disease or first degree relative with autoimmune disease and no known infection with HIV. Additional serum samples were obtained from 51 SLE patients of African American descent (SLE). All SLE subjects met 1997 ACR revised criteria and were enrolled in the prospective SLE cohort at the Feinstein Institute. Serum samples from 80 healthy African American women (AAHC), age 20 to 68, with no use of immunosuppressive agents in the year prior, and 16 Caucasian healthy controls (CHC), age 28 to 50, were purchased from BioreclamationIVT. Serum from 98 unaffected sisters of SLE patients (SIS), age 14–46, was obtained from the Feinstein Institute SisSLE cohort. The SIS cohort included 67 Caucasian, 11 Hispanics, 7 African-Americans and 12 Asians, (one unknown). The study was approved by the Institutional Review Board at the Northwell Health, Manhasset, NY and the Comité d’Ethique de la FMPOS, Bamako, Mali.

### dsDNA ELISA

To detect IgM and IgG anti-dsDNA antibodies, 96-well plates (Costar, 3690, Corning, Kennenbunk, ME) were coated with calf thymus DNA that had been filtered through a 0.45 um cellulose filter (Millipore, Darmstadt, Germany) to remove ssDNA (#2618, Calbiochem, San Diego, CA) at 2μg/ml in PBS. Plates were dry-coated overnight at 370 C and blocked in 3% FBS/PBS for 1 h at room temperature (RT). Plates were washed 3 times and then incubated with serum samples diluted 1:100 in 0.3% FBS/PBS and assayed in triplicate. Plates were washed 5 times in PBS 0.05% Tween, and then incubated with secondary anti-IgM or IgG alkaline phosphatase conjugated antibodies (SouthernBiotech, Birmingham, AL) diluted 1:000 in 0.3% FBS/PBS for 1 h at 370C, washed 3 times, and developed with alkaline phosphatase substrate (Sigma, St. Louis, MO) at room temperature. Plates were read at 405 nm using a PerkinElmer Victor 3 ELISA reader.

### C1q ELISA

Murine monoclonal anti-human C1q (#A201, Quide San Diego, CA) (25 μl/well of 2μg/ml) in PBS was dry-coated into 96-well polystyrene microtiter plates (Costar, 3690, Corning) overnight at 4 °C. Wells were blocked 3% non-fat dry milk with 50ul/well (# M0841, LabScientific Highlands, NJ) in PBS for 4 h at room temperature. After rinsing the wells three times with PBS-0.05%Tween, 25 μl of serum samples diluted in PBS were added to each well. The serum dilutions were obtained by first making a 1:100 dilution and serially re-diluting this solution until it was 1:10,000. Samples were incubated overnight at 4 °C. Wells were then washed 3 times with PBS- Tween. Goat antiserum to human C1q (#A301, Quidel) was diluted 1:1000 in 0.3% non-fat milk in PBS and added (25 μl/well) for 2 h at room temperature. After washing 3 times in PBS- Tween, plates were incubated for 1 h at room temperature with rabbit anti-goat IgG antibody conjugated to alkaline phosphatase (#A-4062, Sigma) diluted in 0.3% non-fat milk in PBS at 1:500. The wells were washed 3 times with PBS-Tween and incubated with 50 μl of alkaline phosphatase substrate (Sigma) in solution (.5 M Na2CO3 and .01 M MgCl2) (check). The absorbance of each well was read at 30 min at 405 nm. The standard curve of purified human C1q was linear in the 2 ng to 250 ng range. Both the standards and serum samples were assayed in triplicate.

### Statistical methods

The primary objective was to compare potential biomarkers of SLE among women grouped by risk for SLE based on race and malaria exposure: healthy Caucasian (CHC) and African American (AAHC) women, African women with past exposure to malaria (MAL), unaffected sisters of lupus patients (SIS), and lupus patients (SLE). Since a high IgG:IgM anti-dsDNA antibody ratio and a low level of C1q are associated with SLE, and a low IgG:IgM anti-dsDNA antibody ratio and high level of C1q are associated with healthy controls, the LRI was calculated by $\frac{IgG}{IgM\ x\ C1q}$ . For this analysis, original measurement units were used and plotted on log axis which resulted in data that were consistent with the usual assumptions of normality and equal variance across groups. One-way analysis of variance was used to compare each of these five markers separately across the groups. Upon finding a significant difference, Tukey’s method of pairwise comparisons was used, separately for each marker, to determine which groups’ means differed from one another on that marker. All statistical tests, including the Tukey test, were performed at the 5% significance level.

## Results

### Anti-dsDNA antibodies

**Fig. 1:**
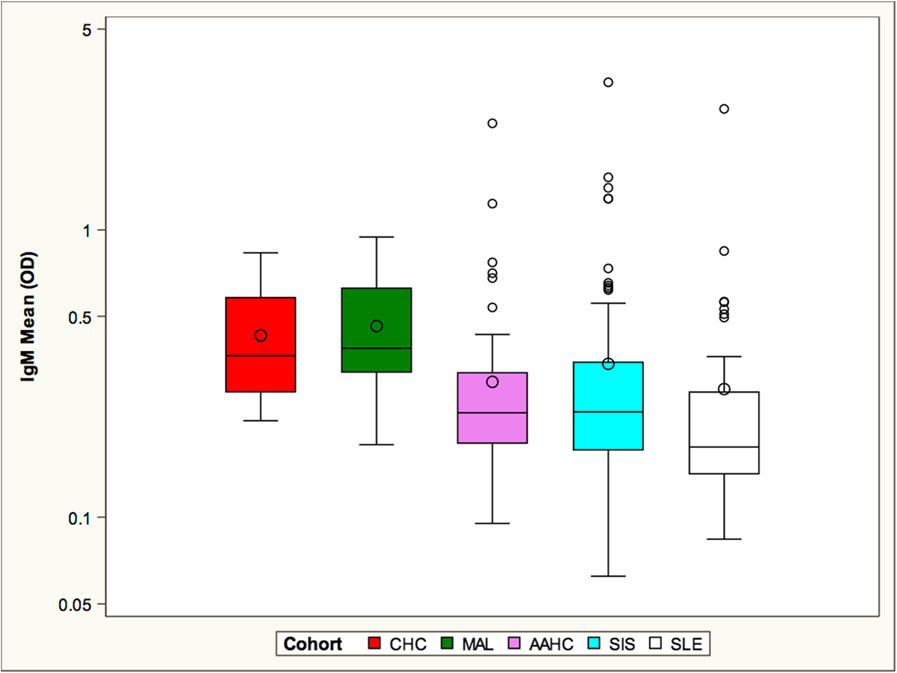
The cohorts are organized in order of the presumed risk from lowest (left) to highest (right). The MAL cohort had the highest mean IgM anti-DNA level, followed by the CHC cohort. The mean of the SIS cohort did not differ from the mean of the AAHC and the SLE cohorts

As IgM antibodies precede the generation of IgG antibodies and protect against SLE onset, we assessed IgM anti-DNA antibodies in all 5 cohorts (Fig. 1). Titers were lowest in the SLE, SIS, and AAHC cohorts. Titers were significantly higher in the CHC cohort and highest in the MAL cohort.

**Fig. 2:**
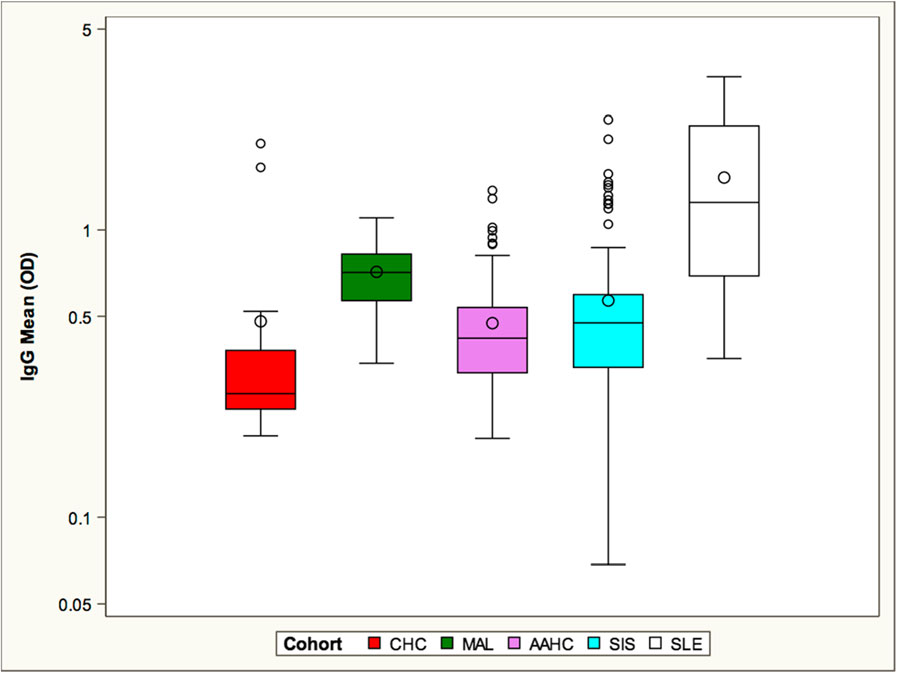
The SLE and MAL cohort had significantly higher mean IgG anti-DNA levels than all other cohorts. The CHC, AAHC and SIS cohorts did not differ from one another and had lower titers than the MAL and SLE cohorts

We next assessed IgG anti-DNA antibodies in all cohorts (Fig. 2). CHC, AAHC and SIS had similar titers of these antibodies. The MAL cohort exhibited significantly increased IgG anti- dsDNA titers and the SLE cohort exhibited the highest titers.

### IgG:IgM ratio

**Fig. 3:**
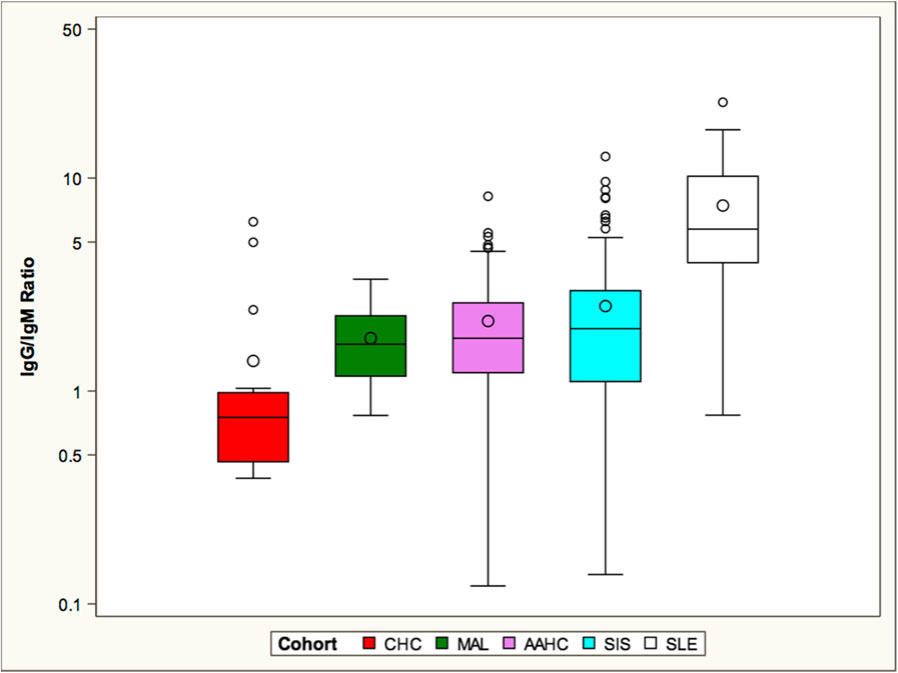
SLE had the highest mean IgG/IgM anti-DNA antibody ratio. The mean ratios of the SIS, AAHC, and MAL cohorts did not significantly differ from each other. The mean ratio for the CHC cohort was significantly lower than all other groups

While significant differences in IgG and IgM anti-DNA titers were present, we reasoned that IgM and IgG antibodies compete for antigen, leading us to ask whether the IgG: IgM ratio was more critical to disease progression than the titer of either alone (Fig. 3). As expected, the SLE cohort had the highest ratio compared to all other cohorts. The MAL, SIS and AAHC cohorts had an intermediate ratio while the CHC cohort had the lowest ratio.

### C1q levels

**Fig. 4:**
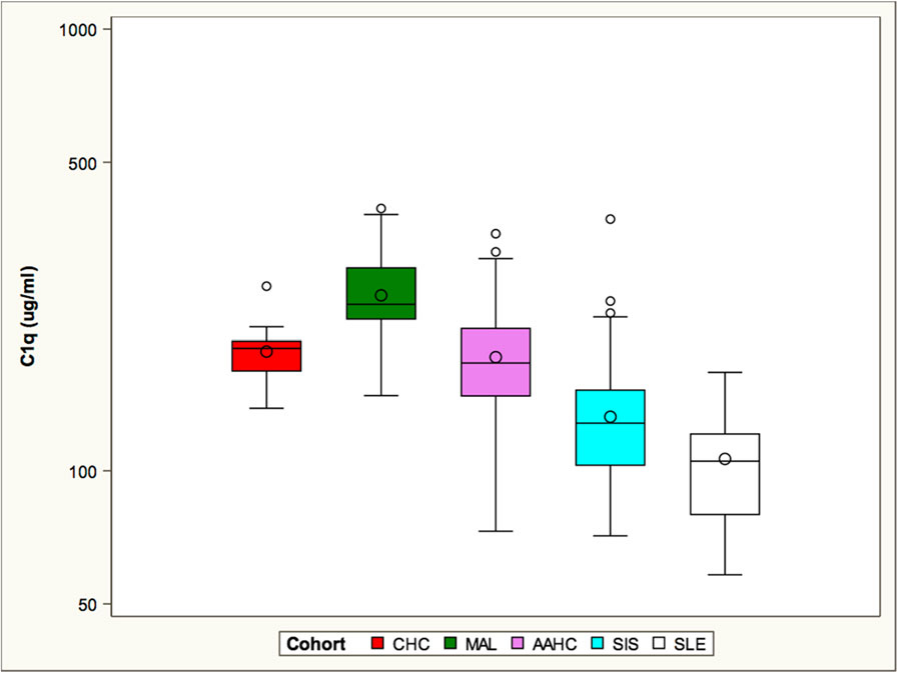
The MAL cohort had the highest mean C1q level. The mean C1q levels of the CHC and AAHC cohorts did not differ from each other. The SIS cohort had a lower mean C1q level and the SLE cohort had the lowest level

C1q levels were assessed in all cohorts (Fig. 4). Not only is C1q deficiency among the greatest risk factors for SLE, but C1q inversely correlates with disease activity (Horak et al. 2006). Anti-C1q antibodies have also correlated with disease activity (Bock et al. 2015). C1q levels were lowest in the SLE cohort, slightly higher but still low in the SIS cohort, intermediate within the CHC and AAHC cohorts and highest in individuals exposed to malaria, the MAL cohort.

### Lupus risk index

**Fig. 5:**
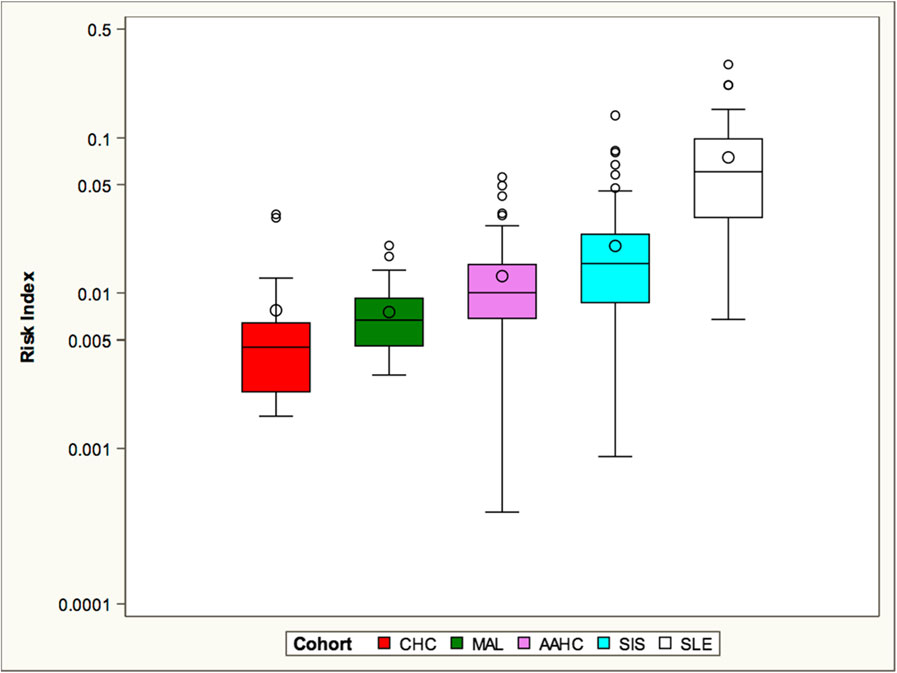
All cohorts were significantly different from each other, except the CHC and MAL cohorts which showed no significant difference

Based on the putative protection conferred by a low IgG/IgM anti-dsDNA antibody ratio and a high C1q level, the LRI was developed to measure propensity for development of SLE for each individual (Fig. 5). The LRI was defined as $\frac{IgG}{IgM\ x\ C1q}$ . The SLE patients exhibited the highest mean LRI, followed by the SIS cohort, and then the AAHC cohort, while the CHC and the MAL cohorts exhibited the lowest LRI.

## Discussion

In this study, we examined serologic markers in 5 cohorts with variable risk for SLE to understand pathways that might predispose to or prevent disease onset. As anticipated, we observed high titers of IgM anti-DNA antibodies in the MAL cohort and high titers of IgG anti-DNA antibodies in the SLE cohort. Analysis of the IgG/IgM anti-DNA antibody ratio showed a high ratio in SLE patients, a low ratio in the CHC cohort and intermediate ratios within the SIS, MAL and AAHC cohorts.

The protective properties of IgM antibodies are known. IgM immune complexes engage C1q which will bind LAIR-1, an inhibitory surface receptor on hematopoietic cells (Son and Diamond 2015). IgM precedes IgG anti-dsDNA antibodies in mouse models of SLE and it has been shown in the *NZB/W* model that administration of IgM anti-DNA antibody will delay onset of disease. IgM, especially pentameric IgM, competes with IgG for antigen and thereby diminishes the load of IgG immune complexes including IgG anti-DNA immune complexes that bind to activating Fc receptors on myeloid cells to initiate an inflammatory cascade. Consistent with the model that IgM is protective against autoimmunity and IgG engages inflammatory pathways, mice genetically engineered to secrete IgG but not IgM will develop SLE (Marshak-Rothstein 2006; Boes et al. 2000). Moreover, *B6.Sle1* mice which carry the *sle 1* risk locus from *NZM* mice produce more antigen-specific IgG and total IgG and exhibit enhanced IgM to IgG class switching (Rahman et al. 2007), suggesting that part of the genetic risk for SLE may include a propensity to high IgG levels.

Malaria exposed individuals harbor anti-nuclear antibodies, some of which cross react with malarial antigens. The ANA pattern in malaria is different from patterns observed in SLE, suggesting fine specificity differences, but anti-DNA antibodies have been reported (Hommel et al. 2014; Hirako et al. 2015). That these anti-DNA antibodies are primarily IgM is consistent with reports of high IgM antibodies in response to malarial infection (Pleass et al. 2016; Czajkowsky et al. 2010). Interestingly, the Fulani population in Mali experiences less severe malarial disease than the Dogon population; IgM anti-malarial titers are higher in the Fulani than the Dogon and may in part account for the less severe disease (Maiga et al. 2013). Why malaria exposure leads to high IgM levels and whether this reflects activation of “innate” B1 or marginal zone B cells or impaired class switching in malaria patients is not known, but may relate to high BAFF levels which are seen in malaria exposed individuals (Scholzen and Sauerwein 2013).

We analyzed serum levels of C1q as low C1q correlates with disease severity and absence of C1q is a strong genetic risk factor for SLE. C1q opsonizes apoptotic cells to remove debris in a non-inflammatory fashion in an IgM-mediated pathway. C1q binds the collagen receptor LAIR-1 through its collagen-like tail to maintain monocyte quiescence and prevent monocyte to DC differentiation (Son et al. 2012). The interaction of C1q with LAIR- 1 prevents activation of endosomal TLRs in DCs by nucleic acid ligands. Finally, C1q blocks the transfer of an IFN signature transfer to healthy PBMCs by SLE serum. Thus, IgM antibodies function in conjunction with C1q to mitigate inflammatory pathways.

As expected, C1q levels were diminished in the SLE, and, to a lesser degree, in the SIS cohort. There was no difference between the CHC and AAHC cohorts. C1q levels were highest in the MAL cohort. Mechanisms increasing serum C1q levels are unknown but C1q is produced by anti-inflammatory M2-like macrophages (Fraser et al. 2015). While these have not been specifically shown to be increased in malaria infection, they are increased by helminthic infections (Fairweather and Cihakova 2009). Elevated C1q may also relate to the binding of IgM to Pfem1, a molecule expressed on the membrane of parasite-infected erythrocytes. The interaction of IgM with Pfem prevents the binding of IgM to C1q and may thus raise levels of soluble C1q (Czajkowsky et al. 2010). Based on the IgG:IgM anti-DNA antibody ratio and the C1q level, we generated an LRI. This score confirmed the known risk of SLE; the highest LRI was present in the SLE cohort. Among the non-SLE cohorts, LRI was highest in SIS followed by AAHC, while the CHC and MAL cohorts exhibited the lowest LRI. Although the MAL cohort exhibited relatively high IgG anti-dsDNA antibody titers, the high IgM anti-dsDNA antibody and C1q levels reduced the LRI. These serologic features may contribute to the protection malaria confers against the development of SLE. Understanding how malaria, even when recurrent, blocks the IgM to IgG switch has important therapeutic implications.

## Conclusion

In summary, we have studied populations with different risk for developing SLE to propose a metric to assess that risk. A risk score is as robust as its components are pathophysiologically relevant. DsDNA IgG, IgM and C1q, which are the components of the LRI that we propose, are known to respond to changes in disease activity. A tool such as this that can predict the risk of developing clinical SLE would be useful to assess the effectiveness of early interventions. Therapy with hydroxychloroquine, for example, delays disease onset (Virdis et al. 2015); we would anticipate that its therapeutic effect would be reflected in the LRI. Longitudinal studies, including in our unique sisters cohort are needed to validate our findings. These observations additionally suggest new therapeutic approaches for the treatment of SLE.

## Abbreviations

AAHC: Healthy African American women
CHC: Healthy Caucasian women
LRI: Lupus risk index
MAL: Women from Mali with a history of malaria infection
SIS: Unaffected sisters of SLE patients
SLE: Systemic lupus erythematosus
